# ROS Accumulation by PEITC Selectively Kills Ovarian Cancer Cells via UPR-Mediated Apoptosis

**DOI:** 10.3389/fonc.2015.00167

**Published:** 2015-07-28

**Authors:** Yoon-Hee Hong, Md. Hafiz Uddin, Untek Jo, Boyun Kim, Jiyoung Song, Dong Hoon Suh, Hee Seung Kim, Yong Sang Song

**Affiliations:** ^1^Gynecological Oncology Laboratory, Cancer Research Institute, Seoul National University College of Medicine, Seoul, South Korea; ^2^WCU Biomodulation, Department of Agricultural Biotechnology, Seoul National University, Seoul, South Korea; ^3^Department of Obstetrics and Gynecology, Seoul National University Bundang Hospital, Seongnam, South Korea; ^4^Department of Obstetrics and Gynecology, Seoul National University College of Medicine, Seoul, South Korea

**Keywords:** unfolded protein response, β-phenethyl isothiocyanate, ovarian cancer, reactive oxygen species, apoptosis

## Abstract

Unfolded protein response (UPR) is crucial for both survival and death of mammalian cells, which is regulated by reactive oxygen species (ROS) and nutrient depletion. In this study, we demonstrated the effect of ROS-accumulation, induced by β-phenethyl isothiocyanate (PEITC), on UPR-mediated apoptosis in ovarian cancer cells. We used ovarian cancer cell lines, PA-1 and SKOV-3, with different p53 status (wild- and null-type, respectively). PEITC caused increased ROS-accumulation and inhibited proliferation selectively in ovarian cancer cells, and glutathione (GSH) depletion in SKOV-3. However, PEITC did not cause any effect in normal ovarian epithelial cells and peripheral blood mononuclear cells. After 48 h of PEITC treatment (5 μM), apoptotic cell death was shown to increase significantly in the ovarian cancer cells and not in the normal cells. The key regulator of UPR-mediated apoptosis, CHOP/GADD153 and endoplasmic reticulum resident chaperone BiP/GRP78 were parallely up-regulated with activation of two major sensors of the UPR [PERK and ATF-6 in PA-1; PERK, and IRE1α in SKOV-3) in response to ROS accumulation induced by PEITC (5 μM). ROS scavenger, *N*-acetyl-*L*-cysteine (NAC), attenuated the effect of PEITC on UPR signatures (P-PERK, IRE1α, CHOP/GADD153, and BiP/GRP78), suggesting the involvement of ROS in UPR-mediated apoptosis. Altogether, PEITC induces UPR-mediated apoptosis in ovarian cancer cells via accumulation of ROS in a cancer-specific manner.

## Introduction

Ovarian cancer is the second most common gynecological malignancy worldwide and fifth leading cause of death in women in the United States (1). About 80% of women are diagnosed at the advanced-stage of the disease and therefore have poor prognosis. The 5-year survival rate is 30% or less for patients with advanced disease (2, 3). This fatality is due to frequent recurrence and resistance to chemotherapy (4–6). Chemoresistant cells are shown to evade cell death signals in some types of malignancies through metabolic alterations and/or by some currently unknown mechanisms (7, 8).

The endoplasmic reticulum (ER) is the major protein-folding site in eukaryotic cells and is constantly involved with protein processing in cancer cells. The accumulation of unfolded or misfolded proteins causes ER stress and activation of the unfolded protein response (UPR), which is crucial for the determination of cell survival or death under conditions of stress (9–11). Under normal circumstances, the UPR carries out its activities using three major sensors, pancreatic ER kinase (PKR)-like ER kinase (PERK), activating transcription factor-6 (ATF-6), and inositol-requiring enzyme 1 (IRE1) in an interactive manner (12). PERK performs dual roles by phosphorylation of eukaryotic initiation factor 2α (eIF2α) and can either induce the pro-apoptotic pathway (13) and/or can form autophagosomes (14). ATF-6 primarily performs a regulatory role through the transcription of ER protein chaperones (15) such as binding of the immunoglobulin protein (BiP), also known as 78 kDa glucose-regulated protein (GRP78) (16). On the other hand, IRE1 with tumor-necrosis factor receptor-associated factor 2 (TRAF2) activates c-Jun N-terminal kinase (JNK) to promote apoptosis (17). Out of the signaling molecules downstream of the UPR sensors, CCAAT/enhancer-binding protein homologous protein (CHOP), also known as growth-arrest and DNA-damage-inducible gene 153 (GADD153), is considered as an essential factor to mediate ER stress-induced apoptosis (18). ER stress-induced autophagy may lead to either cell survival or cell death. As a survival mechanism, autophagy degrades unfolded and misfolded proteins and recycles them. Although, when long-term ER stress overwhelms protein folding capacity, cell death is activated by apoptotic or non-apoptotic means, while autophagy remains as an active process (11, 12, 19). However, reducing the level of ER stress in different metabolic diseases may also have therapeutic potential, stringent activation of ER stress may be helpful in fighting against cancer (20).

Reactive oxygen species (ROS) have recently been considered as central regulators of ER function, especially in UPR signaling in various diseases, including cancer (21). Such ROS-mediated UPR could lead to apoptosis through the activation of PERK. In addition to its canonical function, PERK also physically interacts with mitochondria-associated membrane (MAM) to regulate inter-organellar crosstalk in ROS-induced cell death (22). ROS have a dual role inside cells. In cancer cells, durable ROS determine cell survival by inducing several survival pathways responsible for cell proliferation, apoptosis suppression, cell migration, and invasion, as well as suppression of the immune system. High ROS levels, however, can be toxic to cancer cells and lead to cell death (23). As demonstrated by previous studies, the levels of ROS around threshold could determine cell fate to go death or survival and this could be target for cancer therapy.

Several recent studies suggest that dietary phytochemicals may offer a chemo-preventive effect against many types of malignancies (24). Furthermore, epidemiological evidence showed that there is an inverse relationship between the intake amount of dietary isothiocyanates (ITCs) and cancer risk (25, 26). Among all the ITCs, β-phenethyl isothiocyanate (PEITC) has reached the level of phase 2 clinical trials for lung and oral cancer prevention (27, 28). PEITC is a well-known phytochemical found in its glucosinolate precursor form in cruciferous vegetables such as watercress and broccoli (27, 29). Due to its potential use in preventive medicine and as a cancer therapeutic agent, PEITC has gained the attention and interest of the cancer research community worldwide (27). This particular ITC is a potent generator of ROS (30, 31), and it was previously reported that increased levels of ROS may activate UPR-induced cell death in cancer cells (32). However, PEITC-induced ROS-UPR-mediated killing of ovarian cancer cells has not yet been evaluated.

As such, the present study was conducted to explore the effects of PEITC on UPR-mediated cellular death in ovarian cancer cells.

## Materials and Methods

### Reagents and antibodies

Propidium iodide (PI), *N*-acetyl-*L*-cysteine (NAC), DCFH-DA (6-carboxy-2,7-dichlorodihydrofluorescein diacetate), and PEITC were purchased from Sigma Aldrich (St. Louis, MO, USA) and MTT (3-[4,5-dimethylthiazol-2-yl]-2,5-­diphenyltetrazolium bromide) from Amresco (Solon, OH, USA). Fluorescein isothiocyanate (FITC)-labeled annexin V (Annexin V-FITC) kit was obtained from BD Biosciences Pharmingen (San Diego, CA, USA). Antibodies against ATF-6 and IRE1α were purchased from Cell Signaling Technology (CA), P-PERK, CHOP/GADD153, and poly-ADP ribose polymerase (PARP) from Santa Cruz Biotechnology (CA), BiP/GRP78 from BD Transduction Laboratories^TM^, and GAPDH from AbFrontier (Seoul, South Korea).

### Cell culture

Human ovarian cancer cell lines PA-1 (p53 wild type) and SKOV-3 (p53 null type) were purchased from the American Type Culture Collection (Rockville, MD, USA). PA-1 cells were maintained in MEM and SKOV-3 cells were maintained in RPMI 1640 media. All of the media was supplemented with 10% fetal bovine serum (FBS) (Gibco BRL, Life Technologies, Grand Island, NY, USA), 100 mg/ml penicillin/streptomycin (P/S) (Gibco), and maintained in cell culture dishes (SPL, Seoul, South Korea) at 37°C in a humidified atmosphere with 5% CO_2_.

### Primary culture of human ovarian surface epithelial cells

Human ovarian surface tissues were obtained from non-malignant patients from Seoul National University Hospital (Seoul, South Korea) with Institutional Review Board (IRB) approval (C-1307-008-502). Tissues were gently washed in PBS (Gibco-BRL, Gaithersburg, MD, USA) and placed in a petri dish with surface cortex tissue facing down. Dispase (Gibco, Life Technologies) was then added at a concentration of 2.4 U/ml in PBS and incubated overnight at 4°C. Tissue surfaces were gently scrubbed to collect isolated cells and washed in PBS at 500 g for 4 min in 25°C. The pellet was suspended in 1:1 mixture of MCDB105 (Gibco, Life Technologies) and M199 (Sigma-Aldrich) and cultured in 35 mm dishes as described above. Cells up to passage three were used for the experiments.

### Isolation of peripheral blood mononuclear cells

Isolation of peripheral blood mononuclear cells (PBMCs) from the buffy coat was modified from a previously described method (33, 34). Briefly, the buffy coat was diluted in PBS (1:1) and slowly layered on top of Ficoll-Plaque^TM^–Premium (GE healthcare, Sweden) followed by centrifugation at 400 × *g* for 30 min at 20°C. The PBMC layer was isolated carefully and washed two times with PBS (200 g for 15 min at 20°C). The isolated cells were counted, and then resuspended in RPMI 1640 media supplemented with 10% FBS and 100 mg/ml penicillin/streptomycin. About 2×10^6^ cells/ml were cultured in 5 ml polystyrene round bottom tubes (BD Biosciences) and incubated as described above. This study was carried out in accordance with the protocol approved by IRB of Seoul National University Hospital (C-1307-008-502).

### Measurement of intracellular ROS

2′7′-dichlorodihydrofluorescein diacetate (DCFH-DA; Sigma Aldrich) was used to measure intracellular ROS. DCFH-DA is cleaved by esterase to DCFH, which is oxidized by hydrogen peroxide to form DCF. After exposure to PEITC for 0, 12, and 24 h, the exposed cancer cells were incubated in medium with 25 μM DCFH-DA for a total of 30 min with vortexing every 10 min at 37°C in the dark. Relative fluorescence intensity of DCF (green) was measured using a BD FACS Canto II flow cytometer. DCF fluorescence was measured with excitation at 488 nm and emission at 525 nm. To confirm the generation of intracellular ROS, cells were pretreated for 1 h with 10 mM *N*-acetyl-*L*-cysteine (NAC; a hydrogen peroxide scavenger) as a negative control prior to treatment with PEITC.

### Glutathione assay

Glutathione depletion was measured using glutathione assay kit according to manufacturer instructions (Cayman Chemical, Ann Arbor, MI, USA). Briefly, cells were collected with a rubber policeman and homogenized in cold buffer (50 mM MES buffer, pH 6–7, containing 1 mM EDTA). After centrifugation at 10,000 × *g* for 15 min at 4°C, an equal volume of the MPA reagent (5 g of metaphosphoric acid in 50 ml water, Sigma-Aldrich) was added to the sample and mixed by vortexing for deproteination. The mixture was incubated at room temperature for 5 min, and centrifuged at >2000 × *g* for 2 min. The supernatant was collected for glutathione detection. A standard curve was prepared for the measurement of glutathione level in the samples.

### Cell proliferation assay

The effect of PEITC on cell proliferation was determined by MTT (3-[4, 5-dimethylthiazol-2-yl]-2, 5-diphenyltetrazolium bromide) assay as described previously (35). In brief, SKOV-3 and PA-1 cells were plated onto 96-well plates at a density between 700 and 3000 cells per well, respectively. The cells were cultured for 24 and 48 h with various concentrations of PEITC (0, 1, 2.5, 5, 10, 20, and 40 μM) dissolved in DMSO. Cells were incubated with 50 μl of MTT solution (2 mg/ml) for 3 h at 37°C in the dark. MTT was then removed and cells were solubilized in 100 μl DMSO for 30 min on a shaker. The optical density was measured at 540 nm using a spectrophotometer (Labsystems Multiskan, Labsystems, Helsinki, Finland).

### Detection of apoptotic cells by flow cytometry

Cells were collected by trypsinization with 0.05% Trypsin-EDTA and washed twice with cold PBS. To include the floating cells, prior to trypsinization, culture medium was collected and floaters were spun down by centrifugation in FACS tubes (BD Falcon, CA, USA) at 4°C for 5 min. The cells were then stained with annexin V-FITC and PI according to the manufacturer’s instructions (BD Pharmingen, CA, USA) on ice. Cells were then analyzed by flow cytometry (BD FACSCanto II) within 1 h.

### Western blotting

Western blotting was performed according to methods described in a previous study (35) with minor modifications. In brief, after PEITC treatment, ovarian cancer cells were collected and washed with PBS and trypsinized with 0.05% Trypsin-EDTA. Cell lysates were prepared as described previously (34). Protein quantitative analysis was determined using a BCA assay kit (Thermo Scientific, Hudson, NH, USA). Cell lysates with 20 μg of protein were loaded onto gels and subjected to 6–15% SDS-PAGE. Proteins were then transferred to a nitrocellulose membrane and blocked with 5% skim milk in tris-buffered saline (TBS) containing 0.1% Tween-20 for 2 h. The membrane was then incubated with specific primary antibodies overnight at 4°C, and then incubated with peroxidase-conjugated secondary antibodies. Signals were visualized using a chemiluminescence detection kit (AbFrontier, Seoul, South Korea).

### Statistical analysis

All of the experiments were performed in 3–5 replicates and the data expressed as means ± SEM. Student’s *t*-test and ANOVA with Bonferroni’s *post hoc* test were performed for statistical comparison. GraphPad Prism 5 and statistical software SPSS 20.0 (SPSS Inc., Chicago, IL, USA) were used for the analyses. *P*-value <0.05 was considered statistically significant.

## Results

### PEITC inhibits growth of ovarian cancer cells without inhibiting the growth of normal PBMC cells

**Figure 1:**
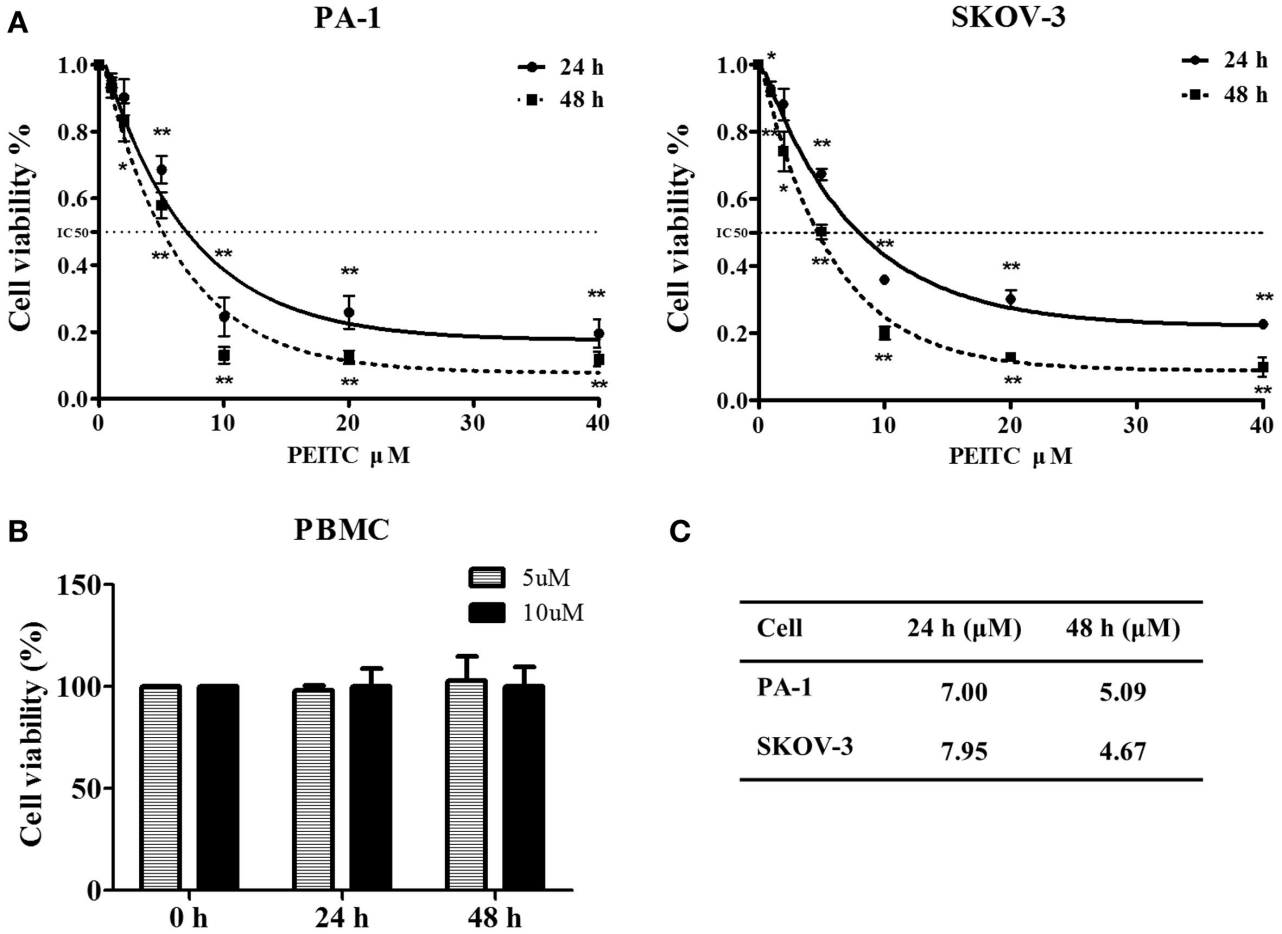
**Inhibitory effect of PEITC on growth of ovarian cancer cells with no effect on that of normal peripheral blood mononuclear cells (PBMCs)**. Ovarian cancer cell lines were incubated with vehicle and various concentrations of PEITC (1, 2, 5, 10, 20, and 40 μM) for 24 or 48 h. PA-1, and SKOV-3 cells were plated onto 96-well plates at a density between 700 and 3000 cells per well, respectively. Normal PBMC were treated with selected concentrations of PEITC (5 and 10 μM). Cellular proliferative activity was measured by MTT assay **(A,B)**. Corresponding IC_50_ value against each cell line after 24 and 48 h of PEITC treatment is presented in the table **(C)**.

Phenethyl isothiocyanate is well recognized for its anti-proliferative activity (28). To determine the effect of PEITC on the growth of normal cells, we performed an MTT assay with concentrations ranging from 1 to 40 μM. Selected doses of PEITC were also tested on normal PBMCs. Both PA-1 and SKOV-3 cells showed significant inhibition of cell proliferation upon PEITC treatment in a dose- and time- dependent manner. Dose–response curve fitting determined the IC_50_ values of PA-1 and SKOV-3 cell lines to be 5.09 and 4.67 μM, respectively, after 48 h of PEITC treatment. To determine the effect of PEITC on normal PBMCs, cells were treated with 5 and 10 μM concentrations of PEITC. We found no significant effect after 24 or 48 h of treatment thereby, suggesting a cancer cell-specific inhibitory effect of PEITC (Figure 1).

### PEITC triggers ROS accumulation in ovarian cancer cells

**Figure 2:**
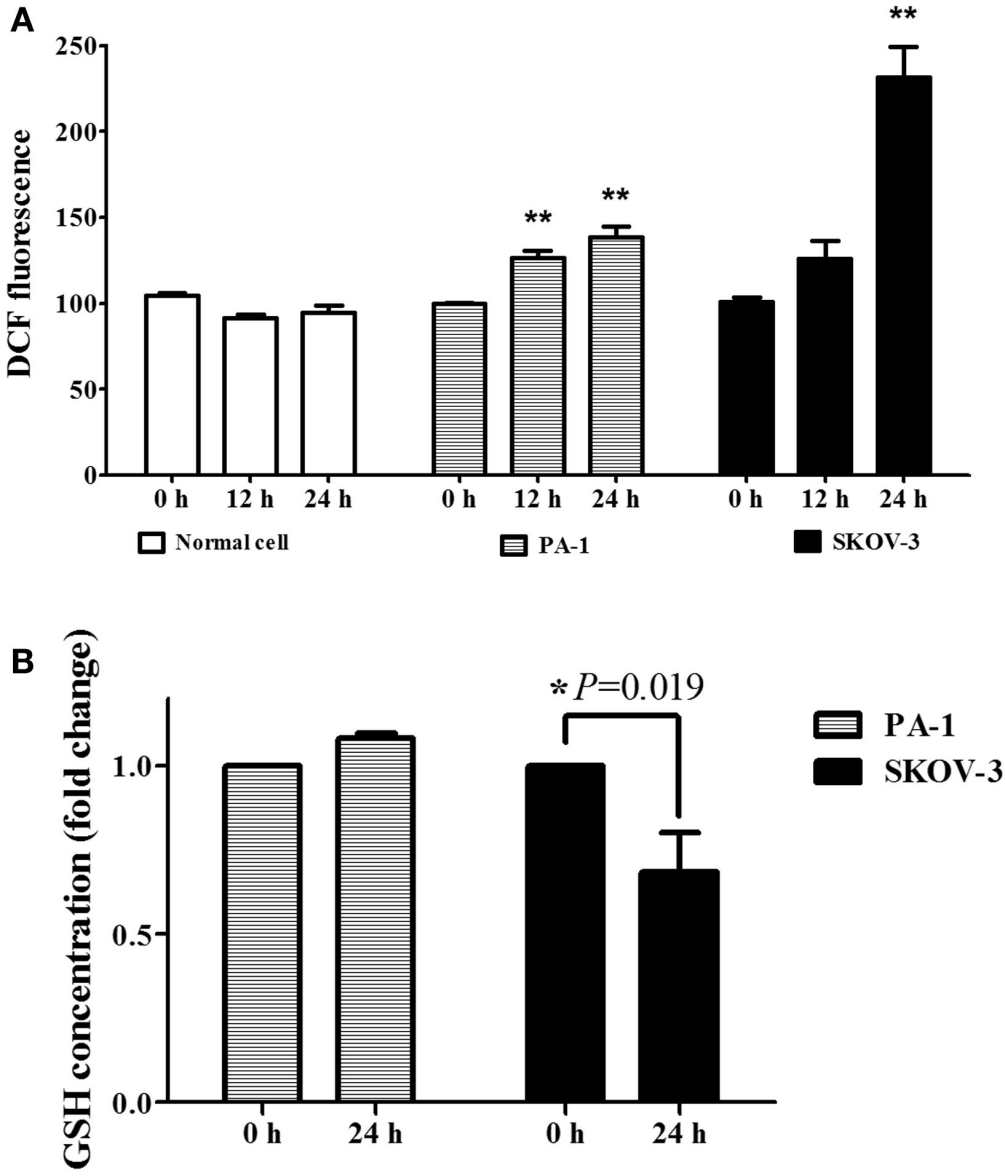
**PEITC causes ROS accumulation only in ovarian cancer cell lines, not in normal OSE (ovarian epithelial cells)**. Normal OSE and cancer cell lines (PA-1 and SKOV-3) were incubated with either vehicle or 5 μM of PEITC for 12 or 24 h and ROS accumulation was detected by flow cytometry using 25 μM DCFH-DA **(A)**. Cells were collected after PEITC treatment for 24 h, and GSH level was measured using glutathione assay kit **(B)**. Values are means ± SEM (**P < 0.01 vs. vehicle control).

We evaluated the effect of PEITC on ROS generation in ovarian cancer cell lines, PA-1 and SKOV-3, by flow cytometry using 25 μM DCFH-DA. We chose to use normal ovarian surface epithelial cells (OSE) as a control. The results showed an increase of ROS in ovarian cancer cells with no effect on normal OSE. In PA-1 cells, the increase of ROS was significantly high at both 12 and 24 h after PEITC treatment. Increased ROS were also observed in SKOV-3 cells; however, it was significant only after 24 h of PEITC treatment. In addition, ROS accumulation was higher at 24 h in SKOV-3 cells compared to that in PA-1 cells (Figure 2A). Then we evaluated GSH level following PEITC treatment. PEITC significantly depleted GSH level in SKOV-3. However, PEITC did not have significant effect on GSH depletion in PA-1 (Figure 2B). Thus, PEITC induces ROS in ovarian cancer cell lines in different mechanism.

### PEITC induces apoptotic cell death in ovarian cancer cell lines

**Figure 3:**
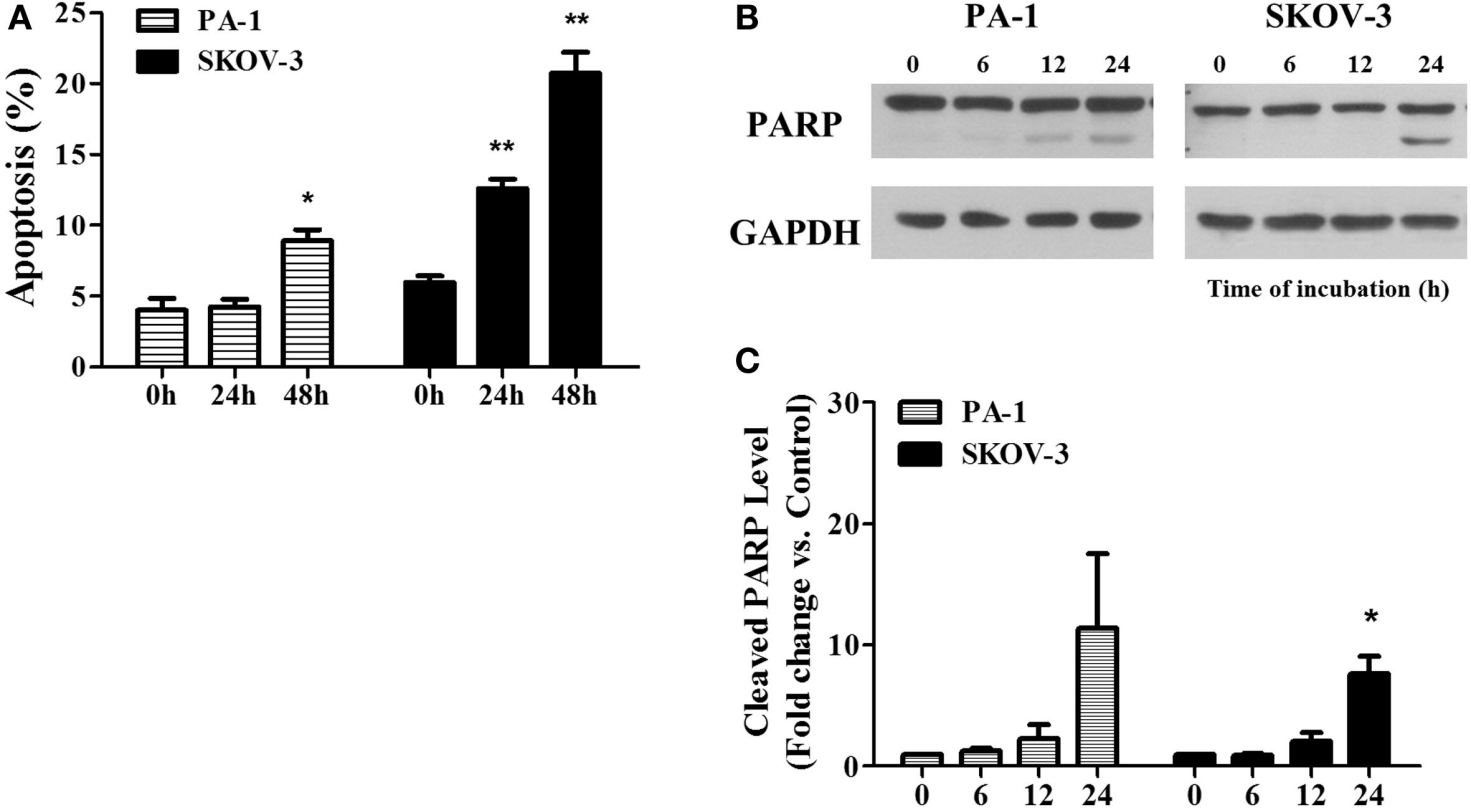
**PEITC induces apoptosis in ovarian cancer cells**. PA-1 and SKOV-3 were incubated with vehicle or 5 μM of PEITC for 24 or 48 h. Apoptotic cell death was measured by Annexin V & PI straining using flow cytometry. Bar graph represents the mean percentage of apoptosis **(A)**. PARP cleavage was detected in PA-1 and SKOV-3 cells after time dependent of PEITC treatment by western blotting **(B)**. Cleaved PARP bands were determined by densitometry of bands using ImageJ software and, statistical analysis was performed by SPSS 20.0 software **(C)**. Values are means ± SEM (*P < 0.05, **P < 0.01 vs. the vehicle control).

In Figure 3, we showed PEITC-induced apoptotic cell death in PA-1 and SKOV-3 cells using the criteria of positive Annexin V-PI staining and PARP cleavage. In PA-1 cells, no significant apoptotic cell death was observed by Annexin V-PI staining after 24 h but, it was significant (up to 10%) after 48 h of treatment. On the other hand, in SKOV-3 cells, we observed more than 10% cell death after 24 h and more than 20% after 48 h (Figure 3A). Western blot analysis revealed that the cleaved fragment of PARP was slightly increased in PA-1 cells after 24 h of PEITC treatment, but it was more prominent in SKOV-3 cells (Figure 3B). These data suggest that upon PEITC exposure, ovarian cancer cell line PA-1is more resistant to apoptotic cell death than SKOV-3.

### ROS scavenger, *N*-acetyl-*L*-cysteine (NAC), reverses PEITC-induced cell death in ovarian cancer cells

**Figure 4:**
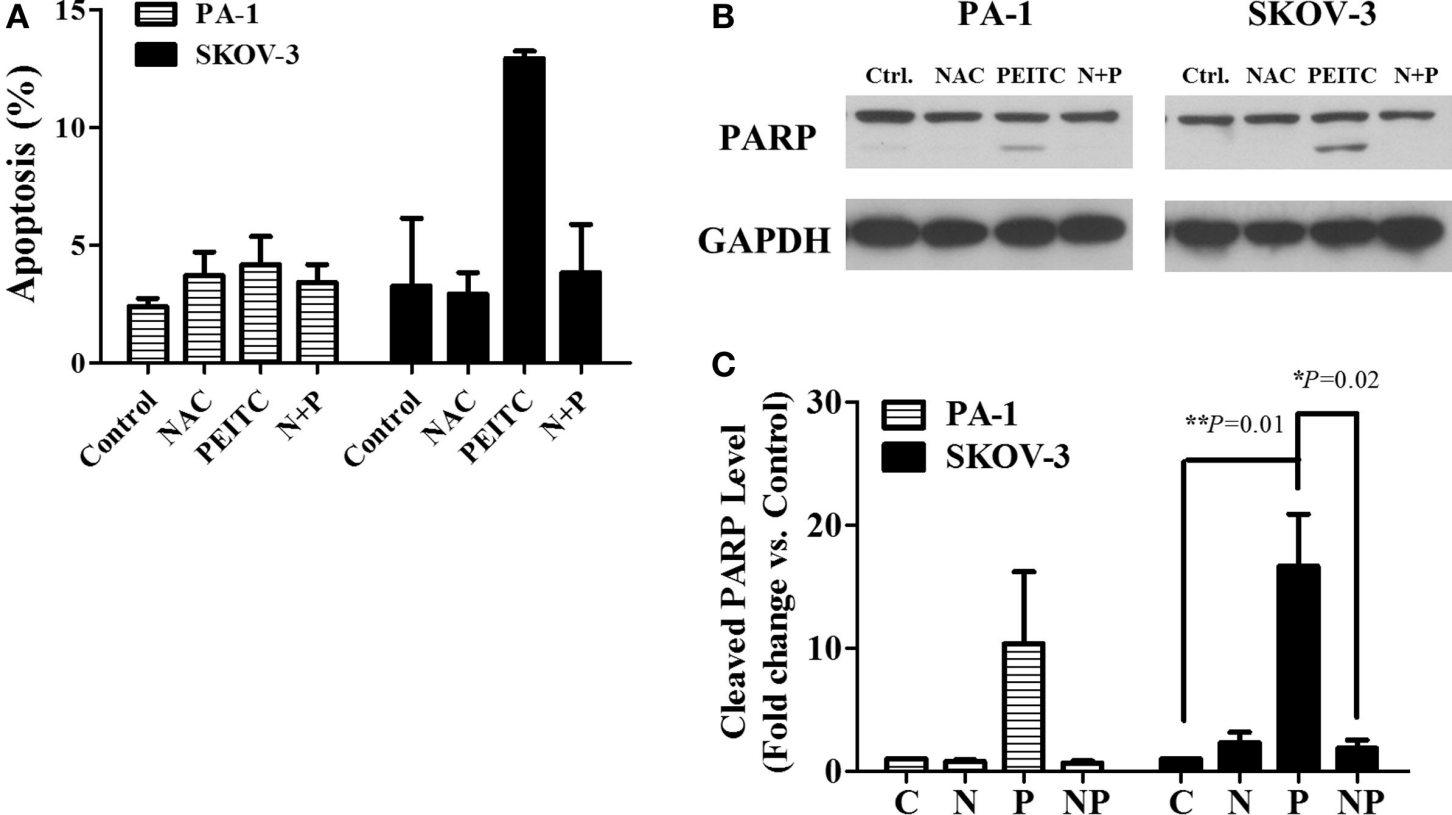
**ROS scavenger, *N*-Acetyl-*L*-Cysteine (NAC), inhibits PEITC-induced apoptotic cell death**. Ovarian cancer cell lines, PA-1 and SKOV-3, were incubated with vehicle, 10 mM NAC, 5 μM of PEITC and NAC with PEITC for 24 h, respectively. During treatment, NAC was added 1 h before PEITC. Apoptotic cell death was detected by annexin V & PI straining using flow cytometry **(A)**. PARP cleavage was detected in PA-1 and SKOV-3 cells after 24 h of PEITC treatment, with or without the addition of NAC by western blotting **(B)**. C, control (DMSO, the vehicle) group; N, NAC-treated group; P, PEITC-treated group for 24 h; N + P, NAC- and PEITC-treated group. NAC was treated for 1 h before 5 μM PEITC treatment. Cleaved PARP bands were determined by densitometry of bands using ImageJ software, and statistical analysis was performed by SPSS 20.0 software **(C)**. Values are means ± SEM (**P < 0.01 vs. the vehicle control).

To determine whether PEITC induced cell death is mediated by ROS or not, we treated cells with the ROS scavenger, NAC, alone or in a combination with PEITC for 24 h. For SKOV-3 cells, PEITC-induced cell death was above 10%, while NAC reduced the cell death to less than 5% (Figure 4A). PARP cleavage was consistent with the Annexin V-PI data, where cleaved PARP was detected upon PEITC treatment, and eliminated with the addition of NAC (Figures 4B,C). In PA-1 cells, cell death determined by Annexin V-PI staining was not significant for any treatment group (Figure 4A). However, although cleaved PARP was observed in PEITC-treated cells alone, it was subsequently abolished in cells pretreated with NAC (Figures 4B,C).

### PEITC induces unfolded protein response, attenuated by NAC, in ovarian cancer cells

**Figure 5:**
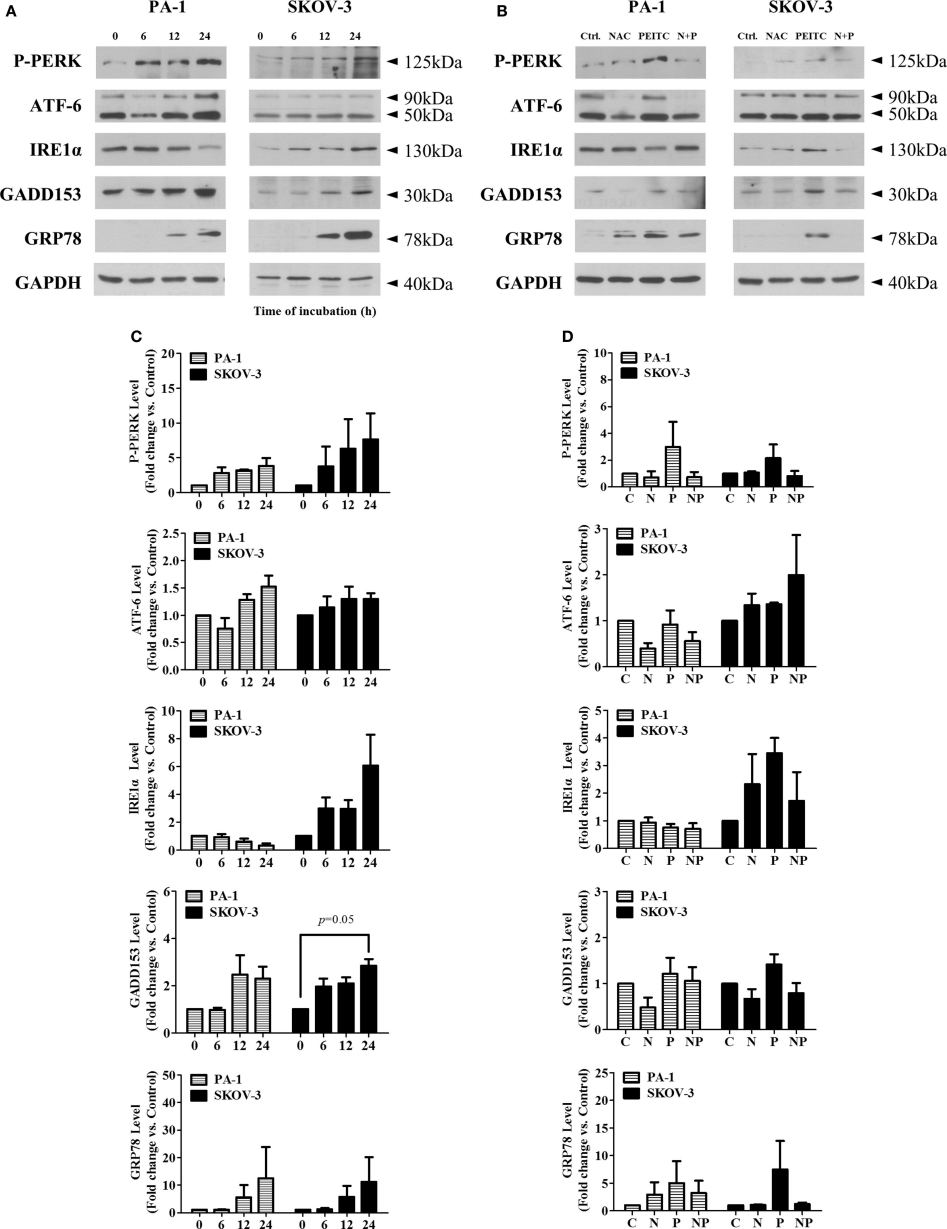
**PEITC induces unfolded protein response with attenuation of UPR by antioxidant, NAC, in PA-1 and SKOV-3 ovarian cancer cells**. Cells were harvested at indicated hours after 5 μM PEITC treatment. Western blotting was performed to detect three arms of UPR (P-PERK, IRE1α, and ATF-6), molecular chaperone GRP78 and GADD153 in **(A)**. With or without 1 h pretreated 10 mM NAC. C, control (DMSO, the vehicle) group; N, NAC-treated group; P, PEITC-treated group for 24 h; N + P, NAC- and PEITC-treated group. NAC was treated for 1 h before 5 μM PEITC treatment **(B)**. P-PERK, IRE1α, ATF-6, GADD153, and GRP78 bands were determined by densitometry of bands using ImageJ software. We analyzed statistical difference of triplicate experiments using ANOVA with Bonferroni’s *post hoc* test and performed using SPSS 20.0 software **(C,D)**. Values are means ± SEM (*P < 0.05, **P < 0.01 vs. the vehicle control).

To determine whether PEITC induces the UPR, we investigated several crucial UPR markers namely P-PERK, ATF-6, inositol-requiring enzyme 1α (IRE1α), CHOP/GADD153, and molecular chaperone BiP/GRP78. In a time-dependent manner, two major arms of UPR (P-PERK and ATF-6) were found to be activated in SKOV-3 cell line (with null type p53), whereas ATF-6 and PERK were activated in PA-1 cells (with wild-type p53). Interestingly, IRE1α was decreased time dependently in PA-1 cells. However, expression levels of downstream target molecules, CHOP/GADD153 and BiP/GRP78, were increased in both cell lines, suggesting an activated ER stress-mediated UPR response with PEITC treatment (Figure 5A). To determine whether this response is ROS related or not, we treated cells with the ROS scavenger, NAC for 24 h (a time point when UPR response was the highest). We found an attenuating effect of NAC for P-PERK, IRE1α, and downstream molecules CHOP/GADD153 and BiP/GRP78 in SKOV-3 cells. In PA-1 cells, NAC treatment also reduced PEITC-induced expression of P-PERK, ATF6, CHOP/GADD153, and BiP/GRP78 (Figure 5B). Western blot bands were quantified and displayed as bar graphs (Figures 5C,D). Altogether, UPR activation by PEITC is likely mediated by ROS in ovarian cancer.

## Discussion

In the present study, PEITC treatment induced activation of UPR and apoptotic cell death in SKOV-3 and PA-1 ovarian cancer cell lines. PA-1 was more resistant to the induction of UPR and apoptosis, than SKOV-3 cells. Addition of antioxidant, NAC, with PEITC suppressed the UPR and apoptosis in both cell lines, especially SKOV-3. PEITC also induced generation of ROS and potent anti-proliferative activity in these cell lines with no significant effect on normal OSE. These results suggest that PEITC may have selectively anticancer effect on ovarian cancer cells through ROS-mediated UPR.

The anti-proliferative effect of PEITC on cancer cells is well documented. It has been observed that PEITC inhibits proliferation in different gynecological malignancies including SKOV-3, TOV-21G, and OVCAR-3 ovarian cancer cells (28, 36). PEITC is a well-tolerated phytochemical for normal cell both *in vitro* and *in vivo* studies (37). In the previous study, IC_50_ value of PEITC for endothelial cells was more than 100 μM, suggesting cancer cell-specific cell death by PEITC (28). PEITC is a well-known ROS inducer in cancer cells without any potential adverse effect on normal cells (30, 37). Consistent with previous studies, there was no significant effect on normal PBMC cells up to 10 μM concentration of PEITC in cell viability and ROS generation assays.

PEITC was used as ROS generator in pancreatic cancer cell line previously (30, 31). It was observed that PEITC treatment generated ROS in normal ovarian epithelial cell (T72) transfected with H-Ras (31). The present study also found increased level of ROS upon PEITC treatment in PA-1 and SKOV-3 cells. However, ROS accumulation was almost two times higher in SKOV-3 than in PA-1. High level of ROS can induce apoptosis, possibly through UPR pathway (38) which might explain the enhanced apoptotic cell death in SKOV-3. The difference of ROS production, UPR pattern, and apoptotic cell death between these two cell lines can be linked to the respective genotype in response to PEITC. In non-small cell lung cancer cells, mutated p53 expressing H1299 cell line was shown to be more sensitive to PEITC than A549 cells with wild-type p53 (39). By contrast, other study observed p53-dependent PEITC-induced apoptotic cell death in transformed mouse epithelial cell, JB6 C1 41 (40). Thus, it remains to be determined whether p53 status is closely linked to the ROS-UPR-mediated apoptosis in cancer cells.

One of the generating ROS mechanisms by PEITC is a depletion of GSH. A role of GSH is known to detoxify ROS within cells. We assessed the GSH concentrations in these cell lines (PA-1 and SKOV-3) after PEITC treatment for 24 h. It showed little increase in PA-1 cell line, while showing the significant decrease in SKOV-3 (*P* = 0.019) (Figure 2B). Accumulating evidence reported that PEITC is rapidly conjugated with GSH, causing a depletion of the GSH pool, and subsequently inducing cell death through oxidative stress (23, 27). The depletion of GSH can be also achieved by targeting its synthesis. After serine synthesis in cells, serine is converted by a glycine intermediate to produce either inosine monophosphate (IMP) to generate purines (GMP and AMP) or GSH (41). Maddocks et al. found that the activated p53 induces transient p21-dependent cell cycle arrest allowing a recovery in GSH pools rather than IMP generation in p53^+/+^ colon cancer cell line. Conversely, these were not observed in either p53^−/−^ or p21^−/−^ isogenic cancer cells (42). It is known that p53 is involved in the regulation of antioxidant mechanism and energy metabolism (43). Under the stressed conditions, p53 induces the expression of glutaminase, resulting in mitochondrial respiration and energy generation. In addition, glutaminase activated by p53 plays a role in antioxidant defense by increasing reduced GSH level, leading to a decrease of ROS level in cells (44). In our study, PEITC increased oxidative stress through ROS accumulation in ovarian cancer cells. However, a different pattern in GSH depletion and ROS generation was shown between PA-1 with p53-wild type and SKOV-3 with p53-null type. Because p53 activated by PEITC may supply a GSH pool to PA-1 (Figure S1 in Supplementary Material), PEITC-induced ROS generation was lower in PA-1 than SKOV-3, and GSH was significantly depleted in SKOV-3 due to an absence of p53. ROS imposed by PEITC could activate ER stress sensors in different mechanism in PA-1 and SKOV-3, contributing to induction of apoptosis. GSH–ROS antioxidant defense mechanism seems to contribute to different sensitivity of PEITC in ovarian cancer cells.

Phenethyl isothiocyanate showed apoptotic cell death both in PA-1 and SKOV-3 cell lines. These results are in agreement with a pervious study where PEITC induced apoptotic cell death in these cell lines (32). However, in the present study, the apoptotic effect of PEITC was less prominent on PA-1 than on SKOV-3, suggesting comparative resistance of PA-1 to PEITC-induced apoptosis. This might be p53 related signaling pathway, the important one against cellular stress such as oxidative and DNA damaging stresses. The apoptotic cell death in these cell lines was reversed by the ROS scavenger, NAC. A number of studies are consistent with our results, ROS mediated cell death in many types of cancer cells (37, 45, 46).

We demonstrated the first evidence that PEITC-induced ROS activates UPR-mediated cell death in ovarian cancer cells. In several studies, PEITC has been shown to generate ROS (30, 31) triggering ER stress with subsequent UPR (11, 19, 38, 47). In the present study, upon PEITC treatment, BiP/GRP78, an ER stress marker, increased in a time-dependent manner in both cell lines. In SKOV-3, two sensors of UPR (P-PERK, IRE1α) became active with the upregulation of CHOP/GADD153. In PA-1, ATF-6, and PERK of UPR were activated, the CHOP/GADD153 was induced as early as 12 h after PEITC treatment. CHOP/GADD153 is an important pro-apoptotic transcription factor closely associated with UPR (48, 49), especially P-PERK-eIF2α pathway (49). Thus, it could be explained that increased level of CHOP/GADD153 was observed in PA-1 and SKOV-3. NAC treatment along with PEITC reduced the expression of UPR sensors (P-PERK and IRE1α in SKOV-3, P-PERK and ATF-6 in PA-1) with decrease of CHOP/GADD153 expression in both cell lines. All these results taken together, the potential role of PEITC-induced ROS could play a critical role in UPR-mediated apoptosis of these ovarian cancer cells (Figure 5B).

In conclusion, this study strongly demonstrated that PEITC can cause cellular death through ROS-driven UPR activity. It induced apoptosis selectively in ovarian cancer cells and inhibited cellular proliferation along with the generation of ROS. Also, NAC treatment attenuated this effect suggesting that the response to the metabolic stress by PEITC could be regulated by ROS in ovarian cancer. Thus, this study showed the cancer-specific effect of PEITC on ovarian cancer cells. ROS and ER stress seem to be responsible for anticancer effects of PEITC on cancer cells and could be targeted for selective therapeutic strategy.

## Conflict of Interest Statement

The authors declare that the research was conducted in the absence of any commercial or financial relationships that could be construed as a potential conflict of interest.

## Supplementary Material

The Supplementary Material for this article can be found online at http://journal.frontiersin.org/article/10.3389/fonc.2015.00167

## References

[1] Jemal A Siegel R Xu J Ward E. Cancer statistics, 2010. CA Cancer J Clin (2010) 60(5):277–300. 10.3322/caac.20073 20610543

[2] Ozols RF. Update on the management of ovarian cancer. Cancer J (2002) 8(Suppl 1):S22–30. 12075699

[3] Classe JM Fontanelli R Bischof-Delaloye A Chatal JF. Ovarian cancer management. Practice guidelines for nuclear physicians. Q J Nucl Med Mol Imaging (2004) 48(2):143–9. 15243409

[4] Bao S Wu Q McLendon RE Hao Y Shi Q Hjelmeland AB Glioma stem cells promote radioresistance by preferential activation of the DNA damage response. Nature (2006) 444(7120):756–60. 10.1038/nature05236 17051156

[5] Kajiyama H Mizuno M Shibata K Kawai M Nagasaka T Kikkawa F. Extremely poor postrecurrence oncological outcome for patients with recurrent mucinous ovarian cancer. Int J Clin Oncol (2014) 19(1):121–6. 10.1007/s10147-013-0522-0 23392995

[6] Ozols RF Recurrent ovarian cancer: evidence-based treatment. J Clin Oncol (2002) 20(5):1161–3. 11870155 10.1200/JCO.2002.20.5.1161

[7] Suh DH Kim MK No JH Chung HH Song YS. Metabolic approaches to overcoming chemoresistance in ovarian cancer. Ann N Y Acad Sci (2011) 1229:53–60. 10.1111/j.1749-6632.2011.06095.x 21793839

[8] Vathipadiekal V Saxena D Mok SC Hauschka PV Ozbun L Birrer MJ. Identification of a potential ovarian cancer stem cell gene expression profile from advanced stage papillary serous ovarian cancer. PLoS One (2012) 7(1):e29079. 10.1371/journal.pone.0029079 22272227 PMC3260150

[9] Ron D Walter P. Signal integration in the endoplasmic reticulum unfolded protein response. Nat Rev Mol Cell Biol (2007) 8(7):519–29. 10.1038/nrm2199 17565364

[10] Jager R Bertrand MJ Gorman AM Vandenabeele P Samali A. The unfolded protein response at the crossroads of cellular life and death during endoplasmic reticulum stress. Biol Cell (2012) 104(5):259–70. 10.1111/boc.201100055 22268789

[11] Faitova J Krekac D Hrstka R Vojtesek B Endoplasmic reticulum stress and apoptosis. Cell Mol Biol Lett (2006) 11(4):488–505. 10.2478/s11658-006-0040-4 16977377 PMC6275750

[12] Suh DH Kim MK Kim HS Chung HH Song YS. Unfolded protein response to autophagy as a promising druggable target for anticancer therapy. Ann N Y Acad Sci (2012) 1271:20–32. 10.1111/j.1749-6632.2012.06739.x 23050960 PMC3499662

[13] Harding HP Zhang Y Bertolotti A Zeng H Ron D. Perk is essential for translational regulation and cell survival during the unfolded protein response. Mol Cell (2000) 5(5):897–904. 10.1016/S1097-2765(00)80330-5 10882126

[14] Kouroku Y Fujita E Tanida I Ueno T Isoai A Kumagai H ER stress (PERK/eIF2alpha phosphorylation) mediates the polyglutamine-induced LC3 conversion, an essential step for autophagy formation. Cell Death Differ (2007) 14(2):230–9. 10.1038/sj.cdd.4401984 16794605

[15] Gardner BM Pincus D Gotthardt K Gallagher CM Walter P. Endoplasmic reticulum stress sensing in the unfolded protein response. Cold Spring Harb Perspect Biol (2013) 5(3):a013169. 10.1101/cshperspect.a013169 23388626 PMC3578356

[16] Lee AH Iwakoshi NN Glimcher LH. XBP-1 regulates a subset of endoplasmic reticulum resident chaperone genes in the unfolded protein response. Mol Cell Biol (2003) 23(21):7448–59. 10.1128/MCB.23.21.7448-7459.2003 14559994 PMC207643

[17] Rao RV Castro-Obregon S Frankowski H Schuler M Stoka V del Rio G Coupling endoplasmic reticulum stress to the cell death program. An Apaf-1-independent intrinsic pathway. J Biol Chem (2002) 277(24):21836–42. 10.1074/jbc.M202726200 11919205

[18] Zinszner H Kuroda M Wang X Batchvarova N Lightfoot RT Remotti H CHOP is implicated in programmed cell death in response to impaired function of the endoplasmic reticulum. Genes Dev (1998) 12(7):982–95. 10.1101/gad.12.7.982 9531536 PMC316680

[19] Rutkowski DT Kaufman RJ. A trip to the ER: coping with stress. Trends Cell Biol (2004) 14(1):20–8. 10.1016/j.tcb.2003.11.001 14729177

[20] Lee J Ozcan U Unfolded protein response signaling and metabolic diseases. J Biol Chem (2014) 289(3):1203–11. 10.1074/jbc.R113.534743 24324257 PMC3894306

[21] Malhotra JD Kaufman RJ Endoplasmic reticulum stress and oxidative stress: a vicious cycle or a double-edged sword? Antioxid Redox Signal (2007) 9(12):2277–93. 10.1089/ars.2007.1782 17979528

[22] Verfaillie T Rubio N Garg AD Bultynck G Rizzuto R Decuypere JP PERK is required at the ER-mitochondrial contact sites to convey apoptosis after ROS-based ER stress. Cell Death Differ (2012) 19(11):1880–91. 10.1038/cdd.2012.74 22705852 PMC3469056

[23] Trachootham D Alexandre J Huang P. Targeting cancer cells by ROS-mediated mechanisms: a radical therapeutic approach? Nat Rev Drug Discov (2009) 8(7):579–91. 10.1038/nrd2803 19478820

[24] Guilford JM Pezzuto JM. Natural products as inhibitors of carcinogenesis. Expert Opin Investig Drugs (2008) 17(9):1341–52. 10.1517/13543784.17.9.1341 18694367

[25] Higdon JV Delage B Williams DE Dashwood RH. Cruciferous vegetables and human cancer risk: epidemiologic evidence and mechanistic basis. Pharmacol Res (2007) 55(3):224–36. 10.1016/j.phrs.2007.01.009 17317210 PMC2737735

[26] Tang L Zirpoli GR Guru K Moysich KB Zhang Y Ambrosone CB Intake of cruciferous vegetables modifies bladder cancer survival. Cancer Epidemiol Biomarkers Prev (2010) 19(7):1806–11. 10.1158/1055-9965.EPI-10-0008 20551305 PMC2901397

[27] Gupta P Wright SE Kim SH Srivastava SK. Phenethyl isothiocyanate: a comprehensive review of anti-cancer mechanisms. Biochim Biophys Acta (2014) 1846(2):405–24. 10.1016/j.bbcan.2014.08.003 25152445 PMC4260992

[28] Satyan KS Swamy N Dizon DS Singh R Granai CO Brard L. Phenethyl isothiocyanate (PEITC) inhibits growth of ovarian cancer cells by inducing apoptosis: role of caspase and MAPK activation. Gynecol Oncol (2006) 103(1):261–70. 10.1016/j.ygyno.2006.03.002 16624391

[29] Wu X Zhou QH Xu K. Are isothiocyanates potential anti-cancer drugs? Acta Pharmacol Sin (2009) 30(5):501–12. 10.1038/aps.2009.50 19417730 PMC4002831

[30] Jutooru I Guthrie AS Chadalapaka G Pathi S Kim K Burghardt R Mechanism of action of phenethylisothiocyanate and other reactive oxygen species-inducing anticancer agents. Mol Cell Biol (2014) 34(13):2382–95. 10.1128/MCB.01602-13 24732804 PMC4054319

[31] Trachootham D Zhou Y Zhang H Demizu Y Chen Z Pelicano H Selective killing of oncogenically transformed cells through a ROS-mediated mechanism by beta-phenylethyl isothiocyanate. Cancer Cell (2006) 10(3):241–52. 10.1016/j.ccr.2006.08.009 16959615

[32] Chan DK Miskimins WK. Metformin and phenethyl isothiocyanate combined treatment in vitro is cytotoxic to ovarian cancer cultures. J Ovarian Res (2012) 5(1):19. 10.1186/1757-2215-5-19 22781119 PMC3439343

[33] Schnekenburger M Grandjenette C Ghelfi J Karius T Foliguet B Dicato M Sustained exposure to the DNA demethylating agent, 2’-deoxy-5-azacytidine, leads to apoptotic cell death in chronic myeloid leukemia by promoting differentiation, senescence, and autophagy. Biochem Pharmacol (2011) 81(3):364–78. 10.1016/j.bcp.2010.10.013 21044612

[34] Gwak H Haegeman G Tsang BK Song YS. Cancer-specific interruption of glucose metabolism by resveratrol is mediated through inhibition of Akt/GLUT1 axis in ovarian cancer cells. Mol Carcinog (2014). 10.1002/mc.22227 25307508

[35] Kim SH Song SH Kim SG Chun KS Lim SY Na HK Celecoxib induces apoptosis in cervical cancer cells independent of cyclooxygenase using NF-kappaB as a possible target. J Cancer Res Clin Oncol (2004) 130(9):551–60. 10.1007/s00432-004-0567-6 15197583 PMC12161875

[36] Loganathan S Kandala PK Gupta P Srivastava SK. Inhibition of EGFR-AKT axis results in the suppression of ovarian tumors in vitro and in preclinical mouse model. PLoS One (2012) 7(8):e43577. 10.1371/journal.pone.0043577 22952709 PMC3428303

[37] Xiao D Powolny AA Moura MB Kelley EE Bommareddy A Kim SH Phenethyl isothiocyanate inhibits oxidative phosphorylation to trigger reactive oxygen species-mediated death of human prostate cancer cells. J Biol Chem (2010) 285(34):26558–69. 10.1074/jbc.M109.063255 20571029 PMC2924093

[38] Guan L Han B Li Z Hua F Huang F Wei W Sodium selenite induces apoptosis by ROS-mediated endoplasmic reticulum stress and mitochondrial dysfunction in human acute promyelocytic leukemia NB4 cells. Apoptosis (2009) 14(2):218–25. 10.1007/s10495-008-0295-5 19130236

[39] Pawlik A Szczepanski MA Klimaszewska A Gackowska L Zuryn A Grzanka A. Phenethyl isothiocyanate-induced cytoskeletal changes and cell death in lung cancer cells. Food Chem Toxicol (2012) 50(10):3577–94. 10.1016/j.fct.2012.07.043 22847136

[40] Huang C Ma WY Li J Hecht SS Dong Z. Essential role of p53 in phenethyl isothiocyanate-induced apoptosis. Cancer Res (1998) 58(18):4102–6. 9751619

[41] Tavana O Gu W. The hunger games: p53 Regulates metabolism upon serine starvation. Cell Metab (2013) 17(2):159–61. 10.1016/j.cmet.2013.01.012 23395165 PMC3634368

[42] Maddocks OD Berkers CR Mason SM Zheng L Blyth K Gottlieb E Serine starvation induces stress and p53-dependent metabolic remodelling in cancer cells. Nature (2013) 493(7433):542–6. 10.1038/nature11743 23242140 PMC6485472

[43] Bieging KT Mello SS Attardi LD. Unravelling mechanisms of p53-mediated tumour suppression. Nat Rev Cancer (2014) 14(5):359–70. 10.1038/nrc3711 24739573 PMC4049238

[44] Hu W Zhang C Wu R Sun Y Levine A Feng Z. Glutaminase 2, a novel p53 target gene regulating energy metabolism and antioxidant function. Proc Natl Acad Sci U S A (2010) 107(16):7455–60. 10.1073/pnas.1001006107 20378837 PMC2867677

[45] Wang Y Wei S Wang J Fang Q Chai Q. Phenethyl isothiocyanate inhibits growth of human chronic myeloid leukemia K562 cells via reactive oxygen species generation and caspases. Mol Med Rep (2014) 10(1):543–9. 10.3892/mmr.2014.2167 24788892

[46] Doudican NA Wen SY Mazumder A Orlow SJ. Sulforaphane synergistically enhances the cytotoxicity of arsenic trioxide in multiple myeloma cells via stress-mediated pathways. Oncol Rep (2012) 28(5):1851–8. 10.3892/or.2012.1977 22922937 PMC3981004

[47] Szegezdi E Logue SE Gorman AM Samali A. Mediators of endoplasmic reticulum stress-induced apoptosis. EMBO Rep (2006) 7(9):880–5. 10.1038/sj.embor.7400779 16953201 PMC1559676

[48] Wang XZ Lawson B Brewer JW Zinszner H Sanjay A Mi LJ Signals from the stressed endoplasmic reticulum induce C/EBP-homologous protein (CHOP/GADD153). Mol Cell Biol (1996) 16(8):4273–80. 8754828 10.1128/mcb.16.8.4273 PMC231426

[49] Oyadomari S Mori M. Roles of CHOP/GADD153 in endoplasmic reticulum stress. Cell Death Differ (2004) 11(4):381–9. 10.1038/sj.cdd.4401373 14685163

