# Supplementary material for: ROS Accumulation by PEITC Selectively Kills Ovarian Cancer Cells via UPR-Mediated Apoptosis
Source: Front Oncol. 2015 Jul 28;5:167. doi: 10.3389/fonc.2015.00167 (PMC4517521; doi:10.3389/fonc.2015.00167)
Supplement: Supplementary file 1 [file image_1.pdf]

Supplementary Material

ROS accumulation by PEITC selectively kills ovarian cancer cells via UPR-mediated apoptosis

Yoon-hee Hong [1,2], Md. Hafiz Uddin[1], Untek Jo[1], Boyun Kim[1,2], Jiyoung Song [1,2], Dong Hoon Suh[3], Hee Seung Kim[4] and Yong Sang Song[1,2,4]*

[1]Gynecological Oncology Laboratory, Cancer Research Institute, Seoul National University College of Medicine, Seoul, Korea
[2]WCU Biomodulation, Department of Agricultural Biotechnology, Seoul National University, Seoul, Korea
[3]Department of Obstetrics and Gynecology, Seoul National University Bundang Hospital, Seongnam, Korea,
[4]Department of Obstetrics and Gynecology, Seoul National University College of Medicine, Seoul, Korea

*** Correspondence:** Yong Sang Song, MD, PhD, Department of Obstetrics and Gynecology, Seoul National University Hospital, Yongon-dong, Jongno-gu, Seoul 110-744, Korea, Tel: +822-2072-2822 Fax: +822-762-3599, Email: yssong@snu.ac.kr

Keywords: unfolded protein response, β-phenethyl isothiocyanate, ovarian cancer, reactive oxygen species, apoptosis.

1. Supplementary Figures and Tables

1.1. Supplementary Figures

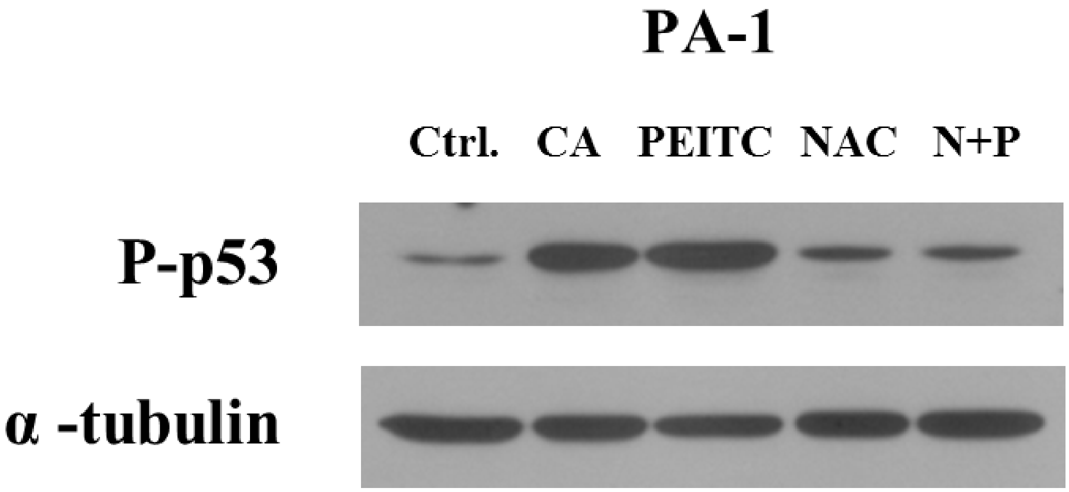

Supplementary Figure 1. PA-1 was incubated with a vehicle, 20 µM cinnamaldehyde (CA), 5 µM of PEITC, 10 mM NAC, and NAC with PEITC for 24 h, respectively. NAC was pretreated for 1 h and co-treated with PEITC for 24 h. Cinnamaldehyde was used as a positive control for checking the activation of p53. Western blotting was performed to detect activation of p53 (P-p53, phosphorylated at Ser15). p53 was activated by PEITC, and attenuated by NAC treatment.
